# Machine Learning Model in Obesity to Predict Weight Loss One Year after Bariatric Surgery: A Pilot Study

**DOI:** 10.3390/biomedicines12061175

**Published:** 2024-05-25

**Authors:** Enrique Nadal, Esther Benito, Ana María Ródenas-Navarro, Ana Palanca, Sergio Martinez-Hervas, Miguel Civera, Joaquín Ortega, Blanca Alabadi, Laura Piqueras, Juan José Ródenas, José T. Real

**Affiliations:** 1 Instituto Universitario de Ingeniería Mecánica y Biomecánica (I2MB), Universitat Politècnica de València, 46022 Valencia, Spain; ennaso@upvnet.upv.es; 2 CIBER de Diabetes y Enfermedades Metabólicas Asociadas (CIBERDEM), Instituto de Salud Carlos III (ISCIII), 28040 Madrid, Spain; esther.benito@hotmail.com (E.B.); balabadi@incliva.es (B.A.); lpiqueras70@hotmail.com (L.P.); jtreal@uv.es (J.T.R.); 3 Endocrinology and Nutrition Service, Clinical University Hospital of Valencia, 46010 Valencia, Spain; a.maria.rodenas@gmail.com (A.M.R.-N.); ana.palanca@gmail.com (A.P.); mi.civeraa@comv.es (M.C.); 4 INCLIVA Biomedical Research Institute, 46010 Valencia, Spain; joaquin.ortega@uv.es; 5 Department of Medicine, University of Valencia, 46010 Valencia, Spain; 6 General Surgery Service, University Hospital of Valencia, 46010 Valencia, Spain; 7 Department of Surgery, University of Valencia, 46010 Valencia, Spain; 8 Department of Pharmacology, University of Valencia, 46010 Valencia, Spain

**Keywords:** obesity, bariatric surgery, RYGB, total weight loss, machine learning, locally linear embedding, predictive model

## Abstract

Roux-en-Y gastric bypass (RYGB) is a treatment for severe obesity. However, many patients have insufficient total weight loss (TWL) after RYGB. Although multiple factors have been involved, their influence is incompletely known. The aim of this exploratory study was to evaluate the feasibility and reliability of the use of machine learning (ML) techniques to estimate the success in weight loss after RYGP, based on clinical, anthropometric and biochemical data, in order to identify morbidly obese patients with poor weight responses. We retrospectively analyzed 118 patients, who underwent RYGB at the Hospital Clínico Universitario of Valencia (Spain) between 2013 and 2017. We applied a ML approach using local linear embedding (LLE) as a tool for the evaluation and classification of the main parameters in conjunction with evolutionary algorithms for the optimization and adjustment of the parameter model. The variables associated with one-year postoperative %TWL were obstructive sleep apnea, osteoarthritis, insulin treatment, preoperative weight, insulin resistance index, apolipoprotein A, uric acid, complement component 3, and vitamin B12. The model correctly classified 71.4% of subjects with TWL < 30% although 36.4% with TWL ≥ 30% were incorrectly classified as “unsuccessful procedures”. The ML-model processed moderate discriminatory precision in the validation set. Thus, in severe obesity, ML-models can be useful to assist in the selection of patients before bariatric surgery.

## 1. Introduction

Obesity represents a major public health problem, and its prevalence, along with its comorbidities, is growing rapidly throughout the world [1,2,3,4]. According to data from the World Health Organization, the global obesity rate has nearly tripled in recent decades [5]. Severe obesity carries an even higher risk of cardiovascular disease and overall mortality, creating additional challenges that require specialized and multidisciplinary teams of healthcare providers [2]. The Study on Nutrition and Cardiovascular Risk in Spain (ENRICA), a cross-sectional study carried out between 2008 and 2012, showed that the prevalence of obesity in adults in Spain was 22.9% [1]. The same work reported a prevalence of morbid obesity of 1.2%. Furthermore, data from previous Spanish studies have shown increasing trends in obesity and morbid obesity rates in the country [1,2].

Conventional dietary and pharmacological strategies have limited efficacy in the treatment of severe obesity. Most patients do not achieve or maintain significant long-term weight loss and therefore require more invasive interventions, such as surgery [3,6,7]. Despite its costs, bariatric surgery (BC) has been considered the treatment of choice for morbid obesity [8]. Cost-effectiveness analyzes have shown that BS can be profitable, the being costs amortized in two to four years [9,10]. Among BS procedures, Roux-en-Y gastric bypass (RYGB) achieves the greatest weight loss within 12 to 18 months, with an average percentage of total weight loss (%TWL) of 30% one year after surgery. However, so far, 15 to 35% of patients who undergo RYGB either fail to achieve adequate weight loss or experience significant weight regain, which often occurs two to five years after surgery [11,12]. As a consequence, the heterogeneity in the results of weight loss after BS underscores the need to identify and analyze the predictive factors that may influence weight loss and weight regain of patients after surgery. In the literature, preoperative predictors of weight loss after surgery have been extensively reviewed [13,14,15,16,17]. Likewise, a study by Wise et al. retrospectively analyzed independent factors related to post-RYGB weight loss. The authors found that female gender, black race, body mass index (BMI) before surgery, and the presence of high blood pressure and diabetes were independently associated with postoperative weight loss [14].

However, BS is not free of complications. Although mortality is very low (<0.5%), there may be both immediate complications after surgery (hemorrhage, dehiscence, etc.) as well as long-term complications such as dumping, gastroesophageal reflux, and nutrient and vitamin deficiency, among others. Nevertheless, the benefit of surgery outweighs the possible complications [7,8,9]. BS procedures are very invasive for patients and costly for healthcare systems. Health information technologies, such as clinical decision support systems (CDSS) capable of estimating in advance the result of an intervention and, therefore, helping in clinical decision-making, are of great interest in this domain [18].

The CDSS include a variety of tools and interventions to support the use of clinical data science in daily medical practices. Within the framework of the CDSS, artificial intelligence (AI) methodologies are applied to the medical field to improve decision-making in health [19]. Machine learning (ML) techniques, a subset of AI, allow machines to learn automatically, identify complex patterns in large data sets, and improve the learning process as new data is received [20,21]. To date, some of the most common medical applications of ML have ranged from cancer diagnosis and treatment to cardiovascular disease, Alzheimer’s disease, visual impairment, depression, and diabetes [22,23,24,25]. ML algorithms have also been successfully applied to analyze large and complex volumes of data from biological and genetic studies [26]. In clinical practice, ML has been used for diagnostic imaging in the areas of radiology and pathology [21,27].

ML techniques applied to BS is a little-studied area. In previous studies, ML techniques have been used to predict the risk of serious postoperative complications and cessation of antidiabetic drugs in patients with type 2 diabetes after surgery [28,29]. However, studies evaluating ML techniques in BS to predict weight loss responses are scarce. The application of ML techniques could contribute to the estimation of the success of RYGP in terms of weight loss after BS, being of potential utility for assisting healthcare professionals in their decision making to better prioritize and stratify severely obese patients who will benefit from BS. In this sense, the main aim of our work was to perform an exploratory study to evaluate the feasibility and reliability of the use of ML techniques to estimate the success or failure in weight loss after RYGP, based on clinical, anthropometric, and biochemical data, in order to identify the factors implicated in poor weight responses in morbidly obese patients.

## 2. Materials and Methods

### 2.1. Subjects

We retrospectively studied 179 consecutive patients with severe obesity who underwent laparoscopic RYGB at the Hospital Clínico Universitario of Valencia (HCUV, Spain) between 2013 and 2017. The criteria for performing RYGB were defined according to the recommendations of the National Institutes of Health (NIH) as follows: BMI > 40 kg/m^2^ or BMI > 35 kg/m^2^ with comorbidities, inability to obtain weight loss despite lifestyle changes implemented for at least five years, knowledge of the risks and benefits of the procedure, age between 18 and 60 years, and without contraindications for BS. All participants included in the study provided their written informed consent to participate in our cohort research study and authorized the use of their anonymized data for analysis.

#### 2.1.1. Data

Patients’ data were obtained from two previous prospective cohort studies conducted in the Department of Endocrinology and Nutrition of the HCUV. Data collection from these studies followed the research protocol developed by the Department’s Nutrition Unit. All data from the 179 patients were included in a common database.

Information on patient demographic details, medical history, and medication use was collected one month prior to surgery, just before starting a standard 4-week very-low-calorie diet (VLCD). Patients followed VLCD for one month prior to RYGB to achieve preoperative weight loss that would facilitate the surgical procedure, reduce perioperative complications, and improve postsurgical outcomes.

Anthropometric measurements and biochemical parameters were also obtained at the beginning of the study and during the follow-up visit one year after the surgical procedure.

A total of 90 variables, continuous and categorical, were recorded at the beginning of the study. Subsequently, 47 more variables were recorded during the follow-up visit one year after surgery.

#### 2.1.2. Input and Outcome Variables

The aim of this study was to identify predictors of success or failure of RYGB in terms of % TWL one year after the surgical procedure. Weight was the only one-year postoperative variable used to fit the model. Baseline data and %TWL (calculated using one-year postoperative weight) were the respective input and outcome variables that were used by the ML algorithm to fit the predictive model. Therefore, our analysis included only those participants for whom weight data was available at one year after surgery. Therefore, of the 179 subjects initially studied, a total of 118 subjects were included in the analysis.

Among the 90 input variables of the study, continuous variables were selected, while categorical or qualitative variables, like dyslipidemia, which can only take one of a limited number of values (yes/no), were adapted to define an order. As an example, the value of 0 was assigned to the absence of dyslipidemia and the value of 1 to its presence. Variables that were not available for all study subjects were eliminated, leaving a total of 60 variables that were included in the analysis (Table 1).

On the other hand, we use the standard procedure shown in the following equation to normalize variables with different units and ranges:

$$y_{v,i}=\frac{x_{v,i}-{\underline{x}}_{v}}{\sigma_{v}}$$

where

$x_{v,i}$ is the value of the variable *v* in patient *i*,

${\underline{x}}_{v}$ is the mean value of the patient variable *v* in the study sample,

$\sigma_{v}$ is the standard deviation of the patient variable *v* in the study sample, and

$y_{v,i}$ is the normalized value of the variable *v* in patient *i*.

The 118 subjects included in the analysis were randomly divided into two groups: (a) the training group, which represented 70% of the sample and was used to train the model using ML and create a predictive model to estimate weight loss one year after surgery, and (b) the validation group, made up of the remaining 30% of the sample, which was used to verify whether the predictive model generated with the training set provided (or not) adequate results.

The outcome variable was percentage of total weight loss (%TWL). According to the Spanish Association of Surgeons (AEC) and the Spanish Society for Obesity Surgery (SECO), the use of percentage of %TWL is recommended as the metric of choice when reporting weight loss outcomes after BS to avoid bias from preoperative BMI [14]. The following equation was used to calculate %TWL:

$$\%TWL=\frac{{VLCD}_{weight}-1\;year\;weight}{{VLCD}_{weight}}\times 100$$

where ${VLCD}_{weight}$ is the basal weight prior to the VLCD.

For classification purposes in our study, the surgical procedure was considered successful if the patient achieved a %TWL ≥ 30. On the contrary, a %TWL < 30 was considered as failure. Currently, there is no clear consensus on which %TWL threshold best represents success after a BS procedure. Therefore, we established our own cut-off value of 30% TWL based on the one-year postoperative results described in most studies published in the field, in addition to the experience of our team with patients undergoing BS [3].

### 2.2. Methods

For data analysis, we applied a ML approach. Algorithms based on artificial neural networks (ANN) are probably the most commonly used type of ML algorithms, but other types of ML algorithms can also be considered. We plan to investigate, in a future study, the most adequate ML algorithm to estimate the success in weight loss after RYGP based on clinical, anthropometric, and biochemical data. In this pilot study, where only a limited set of subjects was available, our objective was not to determine the best ML algorithm. Instead, we sought to find evidence that ML algorithms could be viable candidates for evaluating the success of weight loss after RYGP. This would justify conducting the planned investigation.

The ML algorithm that we have proposed for our investigations considers the Locally Linear Embedding (LLE) technique, previously described in detail [30], as a tool for the evaluation and classification of the main parameters in conjunction with an evolutionary algorithm for the optimization and adjustment of the parametric model [30,31,32]. The defining parameters of ANN models (that also require the use of optimization algorithms during the training phase) are the weights associated with the connections between the artificial neurons. The role of these weights is to increase or inhibit the activation state of the neurons, but they do not possess an interpretable meaning in a conventional sense. However, in the ML algorithm we propose, the parameters that define the model, as will be seen in Section 2.2.2, will indeed be interpretable as they will determine a measure of the influence of each of the input variables on the output value, the %TWL. This interpretability provides a deeper understanding of the model’s functionality. This functionality was a decisive factor in defining the type of algorithm for the pilot study that we present in this work.

We had a data set defined by a high number of variables, that is, defined in a highly dimensional space, which makes its analysis difficult. It is unknown whether these variables (or dimensions) are correlated with each other or with the result of the analysis. In cases like this, principal component analysis—PCA—allows us to extract the most relevant components (dimensions) from the data set [33]. Thus, we can express the data based on a very small number of variables (in most of the different cases from the original variables) that allows us to represent the data in a space of reduced dimension (these techniques are also classified under the term dimensionality reduction—DR—techniques) while preserving most of the information.

We assumed that we did not know a priori if there was any relationship between the 60 selected variables. As we had no evidence that the data set was linear, we used the LLE dimensionality reduction technique. Therefore, using the LLE technique, the original spatial dimensionality of the data (defined by the 60 selected variables that represent the patient information) was reduced to a small number of non-redundant variables that represented the main characteristics of the data set. In general, it will not be possible to interpret the meaning of the variables in the reduced dimensional space. To facilitate the graphic representation of the results obtained by the LLE analysis, we decided that this low-dimensional space would have three variables (w1, w2, and w3) in this pilot study.

#### 2.2.1. Unweighted and Weighted Data

First, we performed the analysis applying the LLE algorithm to the unweighted data. In the case that LLE does not observe any clustering of patients based on whether the RYGB was successful or not, we would apply the analysis for weighted data.

#### 2.2.2. Weighted Data

Each normalized variable $y_{v,i}$ was weighted using the following equation:

$$z_{v,i}=10^{\alpha_{v}}\cdot y_{v,i}=10^{\alpha_{v}}\cdot \frac{x_{v,i}-{\underline{x}}_{v}}{\sigma_{v}}$$

where

$z_{v,i}$ refers to the normalized and weighted value of the variable *v* in patient *i*,

$\alpha_{v}$ refers to the weighting exponent of the variable *v*.

We decided that the exponent α*_v_* for each variable *v* would be assigned a value between −4 and +4. Thus, each variable would have weights between 10^−4^ and 10^4^ (which is between 0.0001 and 10,000). Therefore, variables with lower α would be less important than variables with a higher α.

The values of the exponents α were not known a priori and their variation would modify the results of the LLE technique. Thus, we used the evolutionary optimization algorithm to find the optimal combination of these exponents, for which the application of the LLE technique would yield values of w1, w2, or w3, adequately correlated with postoperative weight loss at one year. Directions w1, w2, and w3 capture the main sources of variability in the data. Therefore, the proposed procedure aims to force alignment of one of these directions with the one-year postoperative weight loss. The other two directions will capture some other unknown source of data variability.

With this procedure, as in the case of other available techniques, the values finally obtained represent a metric of the relevance of each variable on the prediction of the postoperative weight loss. Hence, it is possible to use this information to regenerate the model considering only the subset of the most relevant variables, making the model more applicable.

##### Evolutionary Algorithm Configuration

The following data were used to configure the evolutionary algorithm:

- Number of parameters α: 60;
- Parameters value range: from −4 to +4;
- Objective function: to maximize the correlation coefficient R^2^ between some of the low-dimensional-space variables and patients’ %TWL;
- Individuals (combinations of α values) of each generation: 1200;
- Maximum number of generations considered: 10,000;
- Number of times the process is repeated: 3.

Thus, the total number of parameter combinations considered was as follows:

1200×10000×3=36 millon

To execute the process, one of the α values of the 60 parameters was needed as a reference. To do this, we chose the variable gender and assigned an α value of 0 (α = 0). Thus, the α values of the gender variable were weighted with a value of $10^{0}=1$. Therefore, the number of considered exponents, α, was 59.

#### 2.2.3. Patients in the Low-Dimensional Space after Training

After running the training process, a new representation of the training-set patient was obtained in the reduced coordinate space (w1, w2, w3) with the weighted data.

The representation of the patient values (w1, w2, w3) in the low-dimensional space versus the postoperative %TWL of each patient was also analyzed. The variable w2 was useful for binary classification. Furthermore, as mentioned above, the directions w1, w2, and w3 are uncorrelated and capture the main sources of variability in the data. Therefore, if w2 is at least partially correlated with %TWL, w1 and w3 will capture some other variability of data from unknown sources not correlated with %TWL.

The optimization process is an iterative process with a significant random component. Despite its randomness, if the process is run multiple times, it will always tend to provide the same results if the number of iteration steps is large enough. If that would not be the case, the process should run multiple times. Therefore, as mentioned above, we ran the process three times and selected the third run as it yielded the best results.

The α-parameter optimization algorithm tried to enforce a correlation between any of the variables in the low-dimensional space (w1, w2, w3) and the outcome variable %TWL. Therefore, any input variable that has an effect on the evaluation of the variables w1, w2, and w3 would also be related to %TWL. Thus, a specific variable associated with a low value of the α parameter would not be related to the %TWL and, conversely, a variable associated with a high value of the α parameter would in fact be relevant to the %TWL.

#### 2.2.4. Prediction of Outcomes in the Validation Group

As the previous results showed a certain degree of correlation between the variable w2 of the low-dimensional space and %TWL after BS, the next step was to estimate the value of w2 for patients in the validation group.

We selected a threshold value w2u. Thus, the patients in the validation group with w2 < w2u were diagnosed as “unsatisfactory intervention” and those patients with w2 ≥ w2u as “satisfactory intervention”.

This classification was compared with the actual %TWL of the patients in the validation group to determine the precision and validity of this discrimination procedure. Therefore, patients in the validation group were classified as true positives (when the predictive model correctly classified patients with TWL < 30% as ‘failed intervention’), true negatives (when the predictive model correctly classified patients with TWL ≥ 30% as ‘successful intervention’), false positives (when the predictive model incorrectly classified patients with TWL ≥ 30% as ‘failed intervention’), and false negatives (when the predictive model incorrectly classified patients with TWL < 30% as ‘successful intervention’).

Since the best possible threshold value for w2u was not known, we tested all possible threshold values for w2u for the proposed classification system in the range of values of w2, which takes values between −3.6638 and 2.3480. The receiver operating characteristic (ROC) curve was obtained for this classifier. 

#### 2.2.5. Positioning Estimation in Low-Dimensional Space

To evaluate the performance of the model built on the training group data, the following process was applied to each patient in the validation group. We assumed that if a patient in the validation group was close to several patients in the training group in the 60-dimensional space, it would also be close to the same patients in the training group when represented in the low-dimensional space (w1, w2, w3). Therefore, we first took the 60 variables that describe each patient from the validation group in the multidimensional space. Then, we evaluated the distance between each patient in the validation group and each of the patients in the training group. The distance between any two patients, labeled 0 and 1, was determined according to the following equation:

$$D=\sqrt{\sum_{i=1}^{60}{\left(v_{i_{1}}-v_{i_{0}}\right)}^{2}}$$

in which *v_i_* is the value of the variable (coordinate) *i*-th of the patient in the space of 60 dimensions.

Once the distance was determined, we selected the set of patients in the training group that were closer to each patient in the validation group. For this pilot study, we considered seven patients in each set. Then, considering the multidimensional coordinate space, we determined the influence of the set of patients in the training group on each of the patients in the corresponding validation group. Let ***V****_v_* denote a vector with the values of the 60 variables for a patient in the validation group, and let ***V****_ti_*, with *i* = 1 … 7, denote the vectors with the values of the 60 variables of the 7 patients of the training group closer to the patient of the validation group. Therefore, ***V****_v_* could be written as a function of ***V****_ti_* using the following equation:

$$V_{v}\approx \sum_{i=1}^{7}\beta_{i}\cdot V_{ti}$$

where *β_i_* represents the influence of the patients in the training group surrounding the patient in the validation group.

With the LLE algorithm, we assumed that the above-mentioned level of influence was also maintained in the low-dimensional space. Therefore, we could assume that ***W****_v_* was an unknown vector, not yet evaluated, that would have the values of the three variables (w1, w2, w3) of the patient in the validation group. It was also assumed that ***W****_ti_* was the vector ***V****_ti_*, defined in the multidimensional space, projected in the low-dimensional space of variables (w1, w2, w3). ***W****_ti_* was a known vector since the LLE algorithm estimated it for each patient in the training group. Thus, the value of ***W****_v_* was estimated with an equation similar to the previous one, preserving the influence values *β_i_*:

$$W_{v}=\sum_{i=1}^{7}\beta_{i}\cdot W_{ti}$$

Thus, estimating the value of ***W****_v_* for a patient in the validation group, we obtained the value of w2 for that patient.

## 3. Results

### 3.1. Sample Description

Data from 118 subjects with morbid obesity who had undergone RYGB and completed the one-year follow-up period post-surgery were analyzed. Baseline characteristics of the patients are described in Table 2. Among these, 43 subjects (36%) were men, and 75 (64%) were women. The mean TWL at one year post-surgery was 32.8% for the complete group. Based on the previously established threshold of TWL = 30% to determine the success or failure of BS, 61% of the subjects had successful procedures (TWL ≥ 30%) and 39% had failed procedures (TWL < 30%).

### 3.2. Unweighted Data

**Figure 1:**
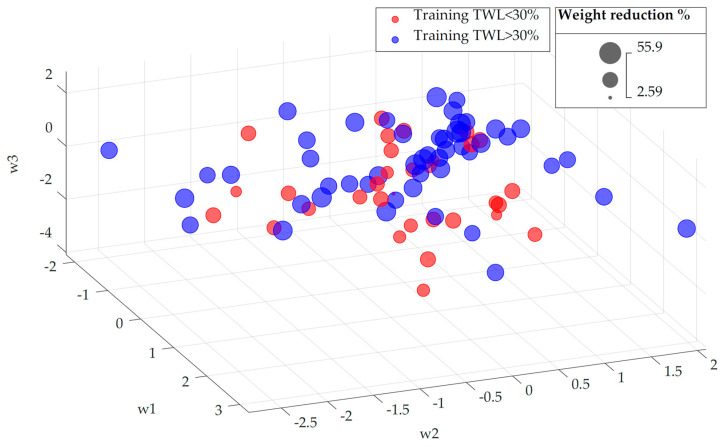
Representation of each training-set patient in the low-dimensional space after applying the LLE algorithm to the unweighted data. Each patient is represented by a bubble whose size is proportional to his/her weight reduction (%TWL). Red bubbles correspond to patients with TWL < 30%, whereas blue bubbles represent patients with TWL ≥ 30%. No grouping can be identified.

The training set after the analysis applying the LLE algorithm to the unweighted data is shown in Figure 1. LLE did not appear to have grouped patients in any special way, and we did not observe any clustering of patients based on whether the RYGB was successful or not.

### 3.3. Weighted Data

**Figure 2:**
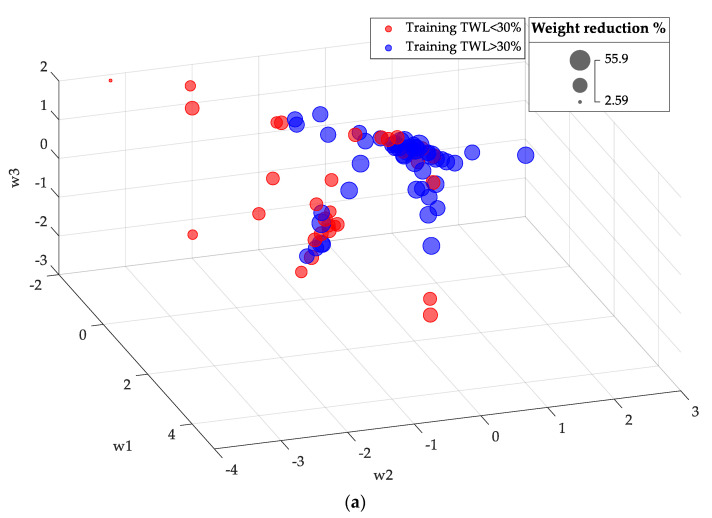

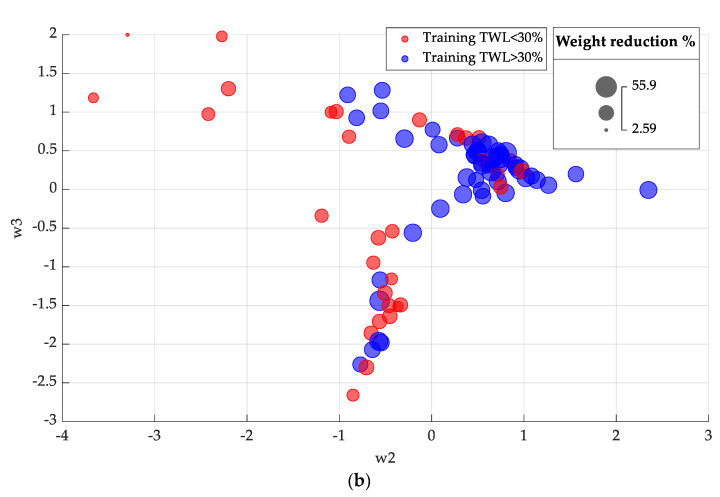
Representation of the training-set patients in the reduced coordinate space (w1, w2, w3) after optimization and weighting: (**a**) perspective view; (**b**) side view. Red bubbles correspond to patients with TWL < 30%, whereas blue bubbles represent patients with TWL ≥ 30%.

The analysis for weighted data is shown in Figure 2. After optimization and weighting, a moderate degree of patient clustering was observed. The lowest values of w2 tended to correspond to patients with a lower %TWL in one year after RYGB.

### 3.4. Patients in the Low-Dimensional Space after Training

**Figure 3:**
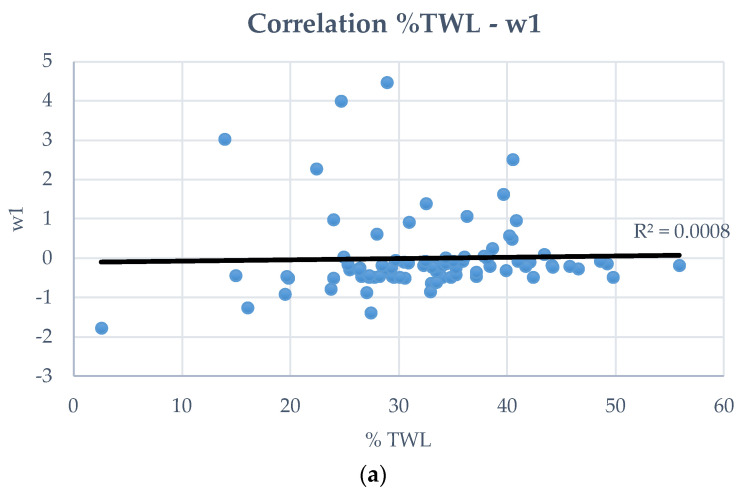

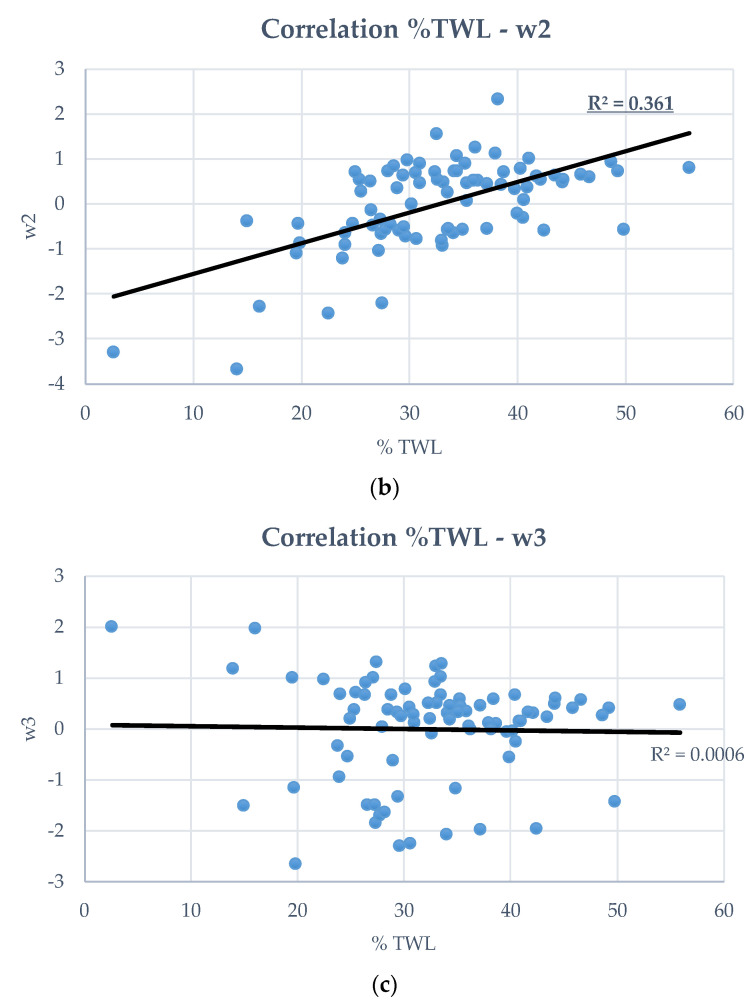
Correlation between low-dimensional space variables (w1, w2, w3) and postoperative percentage of total weight loss: (**a**) w1; (**b**) w2; (**c**) w3. We can observe no correlation between %TWL and w1 and w3 (correlation coefficient < 0.001), while there is some degree of correlation between %TWL and w2 (correlation coefficient = 0.361).

Figure 3 shows the correlation between low-dimensional space variables (w1, w2, w3) and postoperative percentage of total weight loss. We observed a very low correlation between the %TWL and the variables w1 and w3 (correlation coefficient < 0.001), while between the %TWL and the variable w2, the correlation coefficient was 0.361, which shows that there is a certain degree of correlation. Although, with this correlation coefficient, the true %TWL would not be predicted accurately, the variable w2 was useful for binary classification.

### 3.5. Importance of the Variables and %TWL

**Figure 4:**
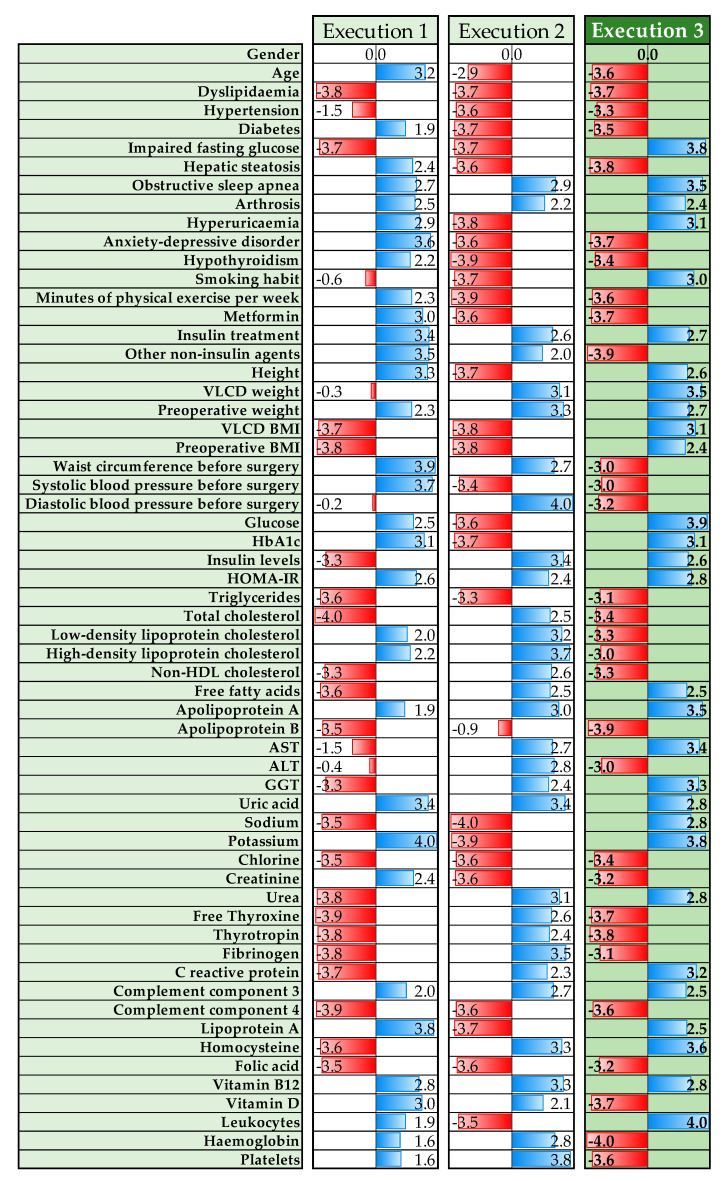
Values of the α parameter for each input variable for the three runs of the optimization algorithm. The α parameters with higher (positive) values are represented with blue horizontal bars whereas red bars are used for the lower (negative) values. The third column shaded in green corresponds to the set of the α parameters finally selected, obtained in the third run.

We ran the optimization process three times and selected the third run as it yielded the best results. Figure 4 shows the values of the α parameters obtained for each run that defined the fitting parameters of the predictive model. 

Some of the α parameters yielded high values in each of the three executed runs. The variables associated with high α values in the three runs were as follows: (1) obstructive sleep apnea (OSA), (2) osteoarthritis, (3) insulin treatment, (4) preoperative weight (after VLCD), (5) insulin resistance index (HOMA-IR), (6) apolipoprotein A (Apo A), (7) uric acid, (8) complement component 3 (C3), and (9) vitamin B12. On the other hand, other α parameters presented low α values in the three runs. Several variables were associated with low α values: (1) dyslipidemia, (2) hypertension, (3) plasma triglycerides (TG), (4) apolipoprotein B (Apo B), (5) chlorine, (6) component 4 of the complement (C4), and (7) folate.

### 3.6. Positioning Estimation in Low-Dimensional Space

**Figure 5:**
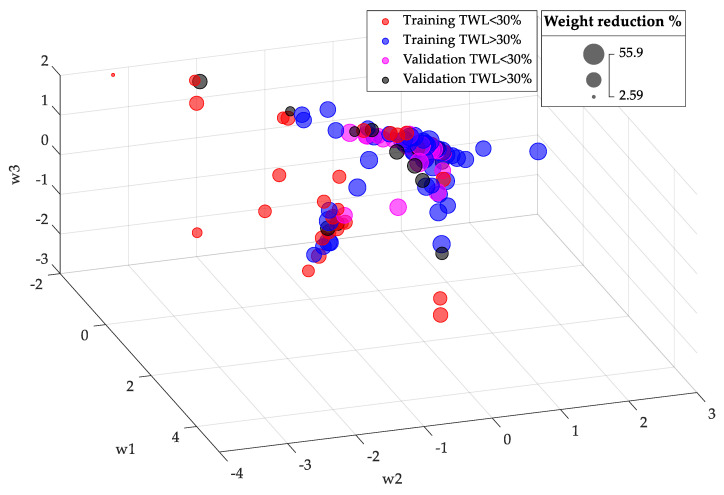
Low-dimensional space position of the training group patients and estimated position of the validation group patients. In red: training group patients with insufficient weight reduction after surgery (TWL < 30%); in blue: training group patients with sufficient weight reduction after surgery (TWL ≥ 30%); in magenta: validation group patients with insufficient weight reduction after surgery (TWL < 30%); in black: validation group patients with sufficient weight reduction after surgery (TWL ≥ 30%).

Figure 5 shows the position within the low-dimensional space of the variables (w1, w2, w3) of the patients in the training group together with the estimated position of the patients in the validation group. Patients from the validation group with insufficient weight reduction after surgery (TWL < 30%) are represented in magenta, while those with adequate weight reduction (TWL ≥ 30%) are represented in black.

### 3.7. Assessment of the Patients in the Validation Group

As described above, we established that the surgical procedure was successful when a patient experienced a TWL weight reduction ≥ 30% after RYGB for one year, while the procedure was considered unsuccessful when the weight reduction was TWL < 30%.

**Figure 6:**
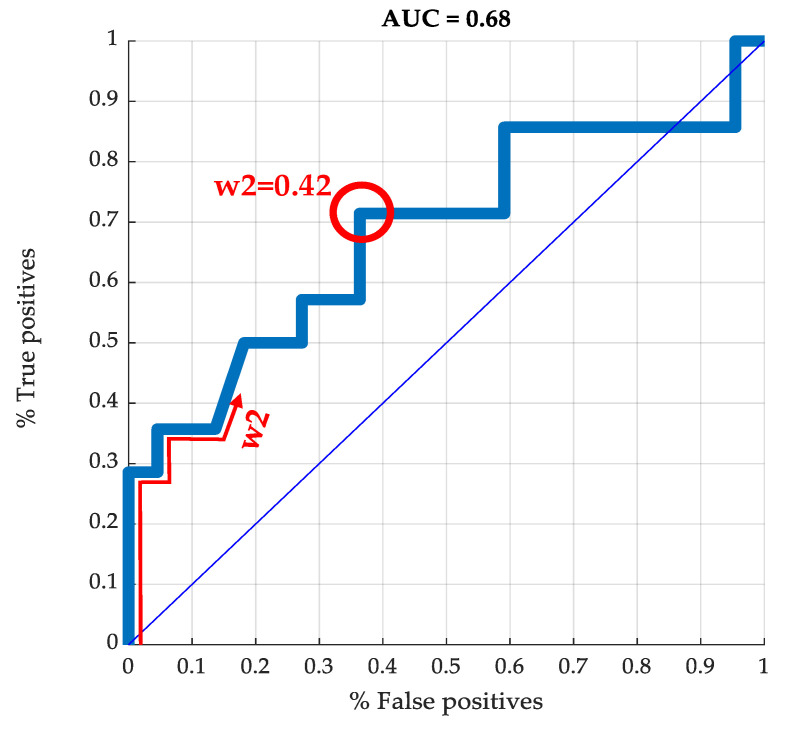
ROC curve obtained for w2u. The point on the ROC curve highlighted in red corresponds to the best threshold value determination for w2u.

Figure 6 shows the ROC curve that was obtained for this classifier (area under the curve (AUC) = 0.68). The point of the ROC curve highlighted in red corresponds to the best threshold value for w2u within the possible values for w2, which corresponds to w2u = 0.4178. Using w2u = 0.4178 and considering the patients in the validation group, we were able to correctly classify 71.4% of patients with TWL < 30% one year after surgery as ‘successful procedures’, although 36.4% of patients with TWL ≥ 30% were incorrectly classified as ‘failed procedures’.

As already indicated, the α values obtained represent a metric of the relevance of each variable in the prediction of the postoperative weight loss. Therefore, we evaluated the ROC curve again, eliminating the variables with α < 2.4 in the 3rd column of Table 2, as they were considered the less relevant variables. The resulting ROC curve evaluated with the remaining 29 variables again produced a value AUC = 0.68.

## 4. Discussion

Data generated by the ML technique related to the field of BS with the purpose of predicting postoperative results are scarce. In this pilot study, we explored the validity of ML to predict RYGB success based on %TWL achieved one year after the procedure. We retrospectively reviewed data from a cohort of severely obese subjects who underwent RYGB at our institution. Based on patient data and using the LLE methodology together with an evolutionary optimization algorithm, we developed a predictive model capable of differentiating between subjects with successful and unsuccessful weight loss responses to surgery.

Previous studies have applied classical statistics that draw population inferences from a sample to predict BS outcomes and post-surgery weight loss [15]. However, ML tools can overcome the limitations of classical statistics when dealing with complex non-linear relationships between a large number of variables [34]. Different BS outcomes have been evaluated in the literature, such as postoperative complications and/or remission of diabetes, through the application of ML techniques [28,29,35]. However, data on the prediction of weight loss after BS using ML are limited. In a cohort of 647 patients (5.5 times larger than our cohort), Wise et al. used artificial neural network (ANN) models to predict one-year weight loss after RYGB. The authors reported a one-year excess body mass index (EBMIL) loss of 73.5% ± 21.5%, corresponding to a TWL of 33.6% ± 8.0%. The prediction generated by the EBMIL 50% ROC curve yielded an AUC for the validation group of 0.83 ± 0.04 [14]. Based on the authors’ prediction model, a web tool was developed to estimate weight loss after RYGB (https://redcap.vanderbilt.edu/surveys/?s=3HCR43AKXR, accessed on 5 May 2024). In our study, we found that the mean TWL at one year was 32.8%, which was similar to the results of the study by Wise et al. However, instead of the ANN model used to develop the predictive model, we applied LLE together with an optimization algorithm. Among the patients in the validation group, our model identified 71.4% of the subjects as true positives and 36.4% as false positives. Interestingly, when we applied the Wise study’s weight loss estimator to our cohort, we found that its estimator overestimated weight loss, classifying 14.3% as true positives of the validation group subjects and 0% as false positives. This finding could be explained by the differences in the protocols of the patients before and after the operation and the follow-up between the two cohorts; therefore, our results cannot be generalized.

In our study, the parameter optimization process was run three times. It was expected to generate the same results in each run, but no consistent results were found because the 10,000 iterations of each run were not enough to achieve full convergence of the process optimization. Nevertheless, the fact that the α parameters associated with some variables took high values in the three runs suggested that these variables are correlated with the postoperative %TWL. However, due to the inherent nature of the applied ML tools, the exact effect of these variables on the post-RYGB %TWL and whether a higher or lower value of these variables would contribute to a more significant %TWL or more reduced could not be determined.

Previous studies have shown a correlation between pre-BS BMI and postoperative weight loss [17,36,37]. In our study, we also found that preoperative weight before VLCD, in addition to postoperative weight after completion of VLCD, were associated with one-year post-RYGB %TWL. Regarding preoperative obesity-related comorbidities, a retrospective study evaluated preoperative clinical factors and long-term weight loss after BS. Investigators found that preoperative insulin use was associated with better postoperative %TWL, whereas hyperlipidemia, higher BMI, and older ages were associated with poorer postoperative weight loss [38]. In a more recent study, Kitamura et al. examined the factors that predict outcomes after BS. The authors found that the presence of a high burden of comorbidities was predictive of worse postoperative weight loss [39]. In the present study, we found that obstructive sleep apnea (OSA), elevated serum urate levels, and osteoarthritis, which are common complications of severe obesity, were associated with postoperative %TWL. We also found that insulin resistance markers, such as HOMA-IR, and insulin treatment before surgery were also related to postoperative weight loss. Furthermore, we found that serum levels of apolipoprotein A (Apo A), the main protein component of HDL cholesterol, were associated with %TWL. In our analysis, the HDL and LDL cholesterol variables showed high values in two of the three runs of the optimization process, thus showing a certain degree of correlation with the result variable %TWL. Both HDL cholesterol and Apo A levels have been clinically associated with insulin resistance [40].

On the other hand, obesity-induced low-grade chronic inflammation has been linked to the pathogenesis of insulin resistance and diabetes [41]. In our work, serum C3 complement concentrations were associated with postoperative weight loss. Complement C3 is an acute phase protein that plays a central role in innate immunity and is produced by the liver, activated macrophage sites of inflammation, and adipocytes [38]. In metabolism, it has been related to insulin resistance, to an increase in the concentrations of free fatty acids, and to adiposity [42,43]. Results from previous studies have shown that serum C3 levels are higher in obese subjects, while C3 levels tend to decrease after weight loss [44]. Thus, inflammatory axes related to the innate immune response or insulin resistance itself may constitute mechanisms by which C3 is related to weight loss.

Interestingly, we also observed an association between preoperative vitamin B12 levels and post-surgical weight loss. Vitamin B12 is a cofactor in homocysteine metabolism, which in turn is a biomarker of oxidative stress [45,46]. Along with oxidative stress, the inflammatory profile drives the chronic low-grade inflammatory state that characterizes obesity and has been linked to insulin resistance. Our findings suggest that the degree of obesity and a set of parameters related to insulin resistance, inflammatory status, and oxidative stress could be involved in the weight response after BS.

Although ML is a powerful modeling tool for predicting outcomes based on data, ML cannot interpret the results. In the present study, we observed an association between %TWL and the low-dimensional space variable w2. However, we could not elucidate the importance of this variable. Nevertheless, our findings on the impact of the examined variables on postoperative weight loss show that the LLE algorithm, in conjunction with the optimization algorithm, generated consistent results from the patient database without introducing more external data during the procedure. Furthermore, the parameters with low values in each of the three runs suggested that these specific variables did not correlate with the outcome variable %TWL.

Regarding the ROC curve obtained in the present study, we must consider that the area under the ROC curve provides a measure of predictive accuracy of a classification system. In the field of BS, a study by Cao Yi et al. used various ML techniques to predict the risk of serious postoperative complications after BS. They found AUC values of 0.56 and 0.58 [28]. In another study, Johnston et al. investigated the cessation of antidiabetic agents in subjects with type 2 diabetes undergoing BS, obtaining AUC values of 0.76 and 0.78 [29]. In our study, we obtained an AUC value in the validation group of almost 0.70. Although it is not directly comparable with Johnston’s study, our result suggests that the precision of the developed prediction model was moderate. However, considering that the present study represents a first exploratory approach in ML techniques to predict post-RYGB weight loss and that the sample size was relatively small (n = 83 for 60 variables), we consider that our results are promising. Our study demonstrates that the use of ML could contribute to improving predictions of the weight loss response in subjects who underwent BS. Weight loss after BS is clinically of great importance. However, the response after surgery is very variable from one patient to another. BS entails a high cost and is not free of complications. Therefore, it is of great interest the possibility of identifying those individuals in whom weight loss will not be adequate. Together our results support the potential utility for assisting healthcare professionals in their decision making to better prioritize and stratify severely obese patients who will benefit from BS. However, more studies are necessary to obtain an adequate algorithm capable of predicting the weight loss response to surgery.

However, our study has some limitations. Although there is growing evidence indicating that socioeconomic and psychosocial factors in combination with eating behaviors can affect BS results [47], in this study, we only had access to clinical, anthropometric, and biochemical data. The incorporation of data on socioeconomic, psychosocial, and diet-related factors could have improved the precision of the classification method developed. Furthermore, although ML algorithms require large amounts of data, the study’s sample size, with only 118 subjects, was relatively small. Several participants were lost to follow-up, while others, who did not live in Valencia, completed the follow-up at their closest hospital. Furthermore, all participants were recruited exclusively from a single institution and underwent the surgical procedure by the same surgical team. This can limit the generality of our model and the external validity of our results. On the other hand, it should be taken into account that the ML algorithm behaves like a black box, thus excluding the interpretation of the results. As described above, the optimization process that was run three times yielded different results each run, a finding that suggests that the optimization process might be in progress and had not reached convergence. Given the limitations of this pilot study, no alternative risk discrimination measures were considered [48] that should have been used to better understand the predictive value of the w2 variable.

## 5. Conclusions

The application of ML techniques showed promising results in estimating the success of RYGP in terms of weight loss one year after BS. Our findings suggest that the degree of obesity, together with the parameters of insulin resistance, inflammation status, and oxidative stress, may influence the weight loss response after BS. ML tools have great potential for CDSS development aimed at assisting healthcare professionals in their decision making to better prioritize and stratify severely obese patients who will benefit from BS.

## Figures and Tables

**Table 1:** Input variables included in the analysis.

Variable Number	Variable Number	Variable Number
1. Gender	21. Pre-VLCD BMI	41. Uric acid
2. Age	22. Preoperative BMI	42. Sodium
3. Dyslipidemia	23. Waist circumference before surgery	43. Potassium
4. Hypertension	24. Systolic blood pressure before surgery	44. Chlorine
5. Diabetes	25. Diastolic blood pressure before surgery	45. Creatinine
6. Impaired fasting glucose	26. Fasting glucose	46. Urea
7. Hepatic steatosis	27. HbA1c	47. Free Thyroxine
8. Obstructive sleep apnea	28. Insulin levels	48. Thyrotropin
9. Arthrosis	29. HOMA-IR	49. Fibrinogen
10. Hyperuricemia	30. Triglycerides	50. C reactive protein
11. Anxiety-depressive disorder	31. Total cholesterol	51. Complement component 3
12. Hypothyroidism	32. LDL-cholesterol	52. Complement component 4
13. Smoking habit	33. HDL-cholesterol	53. Lipoprotein a
14. Minutes of physical exercise per week	34. Non-HDL cholesterol	54.Homocysteine
15. Metformin	35. Free fatty acids	55. Folic acid
16. Insulin treatment	36. Apolipoprotein A	56. Vitamin B12
17. Other non-insulin agents	37. Apolipoprotein B	57. Vitamin D
18. Height	38. AST	58. Leukocytes
19. Pre-VLCD weight	39.ALT	59. Hemoglobin
20. Preoperative weight	40. GGT	60. Platelets

BMI: body mass index; HbA1c: glycated hemoglobin; HDL: high-density lipoprotein; HOMA-IR: homeostatic model assessment of insulin resistance; LDL: low-density lipoprotein; VLCD: very-low-calorie diet.

**Table 2:** Baseline characteristics of the participants included in the analysis and percentage of total weight loss one year after surgery.

	Males (*n* = 43)	Females (*n* = 75)
Age (years old)	45.9 ± 9.2	45.4 ± 9.9
Baseline weight (kg)	144.4 ± 24.6	121.9 ± 19.1 *
Baseline BMI (kg/m^2^)	46.8 ± 6.4	46.7 ± 6.3
Baseline waist circumference (cm)	144.3 ± 13.8	131.3 ± 12.7 *
%TWL	37.8	32.3 *
Systolic blood pressure (mmHg)	144.9 ± 13.8	141.5 ± 18.6
Diastolic blood pressure (mmHg)	89.3 ± 10.2	87.8 ± 8.9
Fasting glucose (m/dL)	104.7 ± 34.2	102.7 ± 38.3
HOMA-IR	6.0 ± 5.5	5.7 ± 4.9
Total cholesterol (mg/dL)	147.9 ± 34.9	166.4 ± 34.5 *
Triglycerides (mg/dL)	123.4 ± 59.1	114.6 ± 43.8
Dyslipidemia, n (%)	28 (65.1)	48 (64)
Hypertension, n (%)	32 (74.4)	42 (56)
Diabetes, n (%)	29 (67.4)	35 (46.6) *
Coronary heart disease, n (%)	5 (11.6)	1 (1.3) *
BMI > 50 kg/m^2^, n (%)	8 (18.6)	18 (24)

Data is shown as mean ± standard deviation. * *p* < 0.05. Abbreviations: BMI: body mass index; HOMA-IR: Homeostasis Model Assessment-Insulin Resistance; TWL: total weight loss.

## Data Availability

The data presented in this study are available on reasonable request from the corresponding author.
